# Incidence rate of sexually transmitted infections among HIV infected patients on long-term ART in an urban and a rural clinic in Uganda

**DOI:** 10.1186/s12889-019-6417-x

**Published:** 2019-01-18

**Authors:** Stephen Okoboi, Barbara Castelnuovo, David M. Moore, Joseph Musaazi, Andrew Kambugu, Josephine Birungi, Mastula Nanfuka, Annelies Van Rie

**Affiliations:** 1 Infectious Diseases Institute; College of Health Sciences, Makerere University, P.O BOX 22418, Kampala, Uganda; 2 Global Health Institute; University of Antwerp, Antwerp, Belgium; 3 Faculty of Medicine and Health Sciences, University of Antwerp, Antwerp, Belgium; 4 British Columbia Centre for Excellence in HIV/AIDS, Vancouver, Canada; 5 University of British Columbia, Vancouver, Canada; 6 The AIDS Support Organization, Kampala, Uganda

**Keywords:** Sexually transmitted infection, Incidence rate, Anti-retroviral therapy, And sexual behavior

## Abstract

**Background:**

HIV immunosuppression increases susceptibility to other STIs and STIs can enhance HIV transmission, reduce CD4 cell count and increase viral load. Co-infections of HIV and STIs may thus reduce the preventive benefits of ART. Little is known about the incidence rate of STIs among long-term patients on ART.

**Method:**

We conducted a secondary data analysis of all patients enrolled in a rural and an urban longitudinal cohort studies who initiated ART between April 2003 and July 2007 followed up to 2016. Patients were screened for STI every three months using “a syndromic and case management approaches”. STI incidence rate, was defined as the number of new cases per population at risk over the follow-up review period. We performed a time-to-event and Kaplan Meier analysis. We used a multivariable Cox proportional hazards regression model to assess for factors associated with STI incidence.

**Result:**

Of 1012 participants, 402 (39.8%) were urban and 610 (60.2%) rural residents. Mean age was 42.8 years (SD 8.5). The total number of follow up time was 44,304 person years. We observed STI incidence rate of 2.1 per 1000 person-years after follow-up. Rural residence (adjusted hazard ratio [aHR] 3.53, 95% CI: 1.95–6.39), younger age (aHR 2.05, 95% CI: 1.02–4.12 for 18–34 years and aHR 1.65, 95% CI: 1.00–2.72 for 35–44 years) were factors associated with higher incidence of STIs. Being male (aHR 0.51, 95% CI: 0.27–0.93) was associated with a lower incidence of STIs.

**Conclusion:**

We found STIs incidence rate of approximately 3 per 1000 person-years among patients on long-term (≥ 4 years) ART followed up-to 3.5 years. Rural and younger persons on ART should be routinely screened for STIs because high incidence of STIs may undo the preventative effects of ART for all.

## Background

HIV-related immunosuppression increases susceptibility to other sexually transmitted Infections (STIs) [1, 2]. Co-infection with STIs facilitates HIV acquisition and transmission by disrupting the integrity of genital mucosa, generating inflammatory responses which recruits HIV target cells, inducing cytokine responses which regulate HIV replication and increase HIV shedding [3, 5, 6, 27, 28]. Genital ulcers in women increases male to female HIV transmission 10 to 50-fold [4]. Although, this data is for people living with HIV not on ART, incident STIs in people on ART may thus reduce the preventive benefits of ART [1]. STI incidence can also serve as a marker of risky sexual behavior [7, 8, 14].

STIs are caused by viral, bacterial, protozoa, fungal and parasitic agents and present as abnormal vaginal or urethral discharges and ulcerative or non-ulcerative genital lesions. Because laboratory diagnosis is complex, expensive, and results in a delayed initiation of treatment, WHO recommended a syndromic and case management approach for developing countries [15, 16]. The Uganda Ministry of Health also recommends a syndromic and case management approaches to STIs among people infected with HIV in public health care facilities. STI related history taking, a physical examination, and clinical algorithms are used to diagnose and management the commonest biological causes of STIs. Where possible, syphilis serologic screening is performed [16–18].

A limited number of studies have evaluated the incidence of STIs among people on ART in sub-Saharan Africa. A South African study found a 14% six-month incidence of STIs measured as treatment seeking for a new STI after initiating ART and observed that individuals diagnosed with an incident STI using a syndromic approach were more likely to have a detectable viral load [12]. Another South African study observed a seven-fold higher rate of STIs after starting ART compared to before ART [9]. In Uganda, a study using STIs syndromic screening algorithms with high cervical swabs for women eligible for intra-uterine contraception where DNA was extracted from the swabs and PCR assay done to detect Neisseria gonorrhea, Trichomonas vaginalis and Chlamydia trachomatis infections found STIs incidence of 11.1% (95% CI: 7.8–14.4) among women on ART [10]. In China, among the 4510 market vendors evaluated for STIs using a syndromic approach by the physician, compared with laboratory tests as a gold standard found a sensitivity of 36.7% among participants reporting STI symptoms and 5.1% among those without an STI symptom [31]. In India, among patients who attended an STI clinic found a sensitivity of genital discharge syndrome for Neisseria gonorrhea at 96% and Chlamydia at 91% while the specificity was low (76 and 72%, respectively). The sensitivity of genital ulcers syndrome for herpes simplex virus-2 and Treponema palladium was 83 and 81% respectively while its specificity was at 99% [32].

While there is some information on STI in the first year(s) of ART, there is a dearth of information on STI incidence in patients on long-term ART in resource-limited settings. Using a syndromic and case management approach, we evaluated the STI incidence rate and assessed factors associated with STIs incidence in patients on long-term ART in an urban and a rural cohort in Uganda.

## Methods

### Study design

We performed a secondary analysis of data from two longitudinal cohorts of individuals on long-term ART: the ART cohort of The Infectious Diseases Institute (IDI) of the College of Health Sciences at Makerere University in Kampala, Uganda [24] and the ART cohort of The AIDS Support Organization (TASO) in Jinja, Uganda [26].

### Study setting

#### The IDI urban ART cohort in Kampala

The adult IDI clinic is a center of excellence for HIV care and treatment [23] located in Mulago Teaching Hospital in Kampala-Uganda. The out-patient clinic has over 30,000 patients ever enrolled and cares for people living in five Kampala municipalities. ART is provided free of charge, initially though the Global Fund and later mainly through the US President’s Emergency Plan for AIDS Relief (PEPFAR).

Following written informed consent, adults (age ≥ 18 years) starting ART between April 2004 and April 2005 were enrolled in a cohort study and followed up for 10 years (hereafter referred to as the urban cohort). ART was started in patients with WHO Stage 4 or CD4 count ≤200 cells/ml, (according to national treatment guidelines implemented at that time), and consisted of stavudine (weight-adjusted, but discontinued in 2013 in Uganda), lamivudine and nevirapine (fixed-dose combination) or zidovudine, lamivudine (fixed-dose combination) and efavirenz. A doctor or clinician and adherence counselor evaluated cohort participants at study enrolment and every three months thereafter while they attended the clinic for ART medication refills [24]. At each study visit, a physical examination was performed and information was recorded about HIV status of the partner(s), social support, sexual history in the past month including STIs screening, diagnosis and treatment. At each clinic visit, the study Doctor asked if the participant experienced any of the syndromic symptoms read to the participant since the last visit, a syphilis serology was done for selected participants based on presenting symptoms. ART was monitored every 6 months through viral load (VL) testing. Participants with a VL ≥1000 copies/ml were offered enhanced adherence counseling and support. Patients with two consecutive viral loads ≥1000 copies/ml were eligible for second-line ART.

#### The TASO rural ART cohort in Jinja

TASO was the first and is one of the largest non-governmental organizations in sub-Saharan Africa (SSA), providing treatment to over 98,000 patients through 11 TASO care facilities in Uganda. Since 2004, TASO Jinja facility has cared for people living with HIV in Jinja district within a radius of 75 km and currently provides ART to over 5000 people. In 2015, A world bank report on development indicators categorized 83.9% of Jinja population as rural. In this study, 81% of the study participants resided outside Jinja municipality.

In June 2012, TASO Jinja initiated a prospective longitudinal cohort study (the Long-Term Outcomes on ART in Uganda Study) of adults who had been receiving first-line ART for a minimum of 4 years (hereafter referred to as the rural cohort) [20, 21]. Between July 1, 2012, and December 31, 2013, all individuals receiving first-line ART for at least 4 years were eligible to participate in the cohort study. From August 2014 until January 2015, enrollment was restricted to patients on ART for at least 4 years with CD4 cell count ≤450 cells/mm^3^ in order to over-sample participants who may have had virologic failure. All participants continued to receive routine comprehensive HIV care from TASO service providers. All participants received a VL test at enrollment. Participants with a VL ≥1000 copies/ml were offered enhanced adherence counseling and VL was re-assessed after three months.

Patients were followed up for an additional 3–3.5 years. Every six months, cohort participants completed an interviewer-administered standardized questionnaire (adapted from the HAARP study) [25] to collect behavioral and treatment outcomes. Clinical care was provided by doctors and clinicians and included STI syndromic screening and management. At each clinic visit the study nurse asked if the participant experienced lower abdominal pain with abnormal vaginal discharge, sores or pus in the penis and vagina since the last visit. and a syphilis serology done for selected participants based on symptoms. Laboratory monitoring included CD4 counts and VL every 6 months. CD4 counts were performed at the Jinja referral hospital regional laboratory. Plasma samples were stored in liquid nitrogen and shipped to the Medical Research Council/Uganda Virus Research Institute (MRC/UVRI) laboratory in Entebbe, Uganda for VL testing and resistance assays.

### Data management and statistical analysis

The urban cohort data were directly collected into an electronic medical record by medical doctors who provided clinical care. Data were periodically validated by a senior Data Manager as part of routine quality control checks. The rural cohort data were double entered using Epi-Info and imported into Microsoft Access for data management and storage. Laboratory test results were transmitted electronically from the MRC laboratory to the data center at TASO Jinja and manually entered into the study database.

For this analysis, data extracted from TASO and IDI database included socio-demographic information, ART start date, ART regimen, sexual behavior, ART adherence at enrolment and at subsequent clinic visits. Clinical data at enrollment and during follow up including information on STI syndromic symptoms and management, CD4 cell counts, and viral load testing were also extracted. Risky sexual behavior was defined as sexual intercourse with ≥2 partners or inconsistent or no condom use in previous 6 months.

In this analysis, baseline was defined as the time of enrollment into the cohort (rural cohort) or the 4-year visit on ART (urban cohort). We described baseline participant characteristics overall and by gender and cohort (urban and rural) using means and frequencies.

The primary outcome of interest was STI incidence rate, defined as the number of new diagnoses per study population at risk during the follow-up (up to additional 3.5 years for both cohorts taking time of enrollment into the cohort (rural cohort) or the 4-year visit on ART (urban cohort) as the baseline. The primary endpoint (STI diagnosis) was defined as syndromic diagnosis (urethral discharge, genital ulcer disease, abnormal vaginal discharge) and a laboratory diagnosis of syphilis. We focused on first STI during follow, only 0.9% of study participants had more than one STI diagnosed during follow up. In time-to-event analysis, all participants contributed person-time from baseline until occurrence of event (STI diagnosis), death, lost to follow up, and transferred out, withdrawal of consent or completion of 3.5 years of follow up, whichever occurred first. A Kaplan-Meier analysis was performed to estimate time to first STI. Using a Cox proportional hazards regression model, we evaluated for associations between STIs and covariates (urban/rural residence, age, CD4 cell count, viral load, gender, education level, marital status, occupation, and time on ART). Covariates were included in the initial Cox model based on prior knowledge and results of the bivariable association and removed using a backward elimination procedure to identify factors independently associated with STI incidence.

## Results

**Fig. 1:**
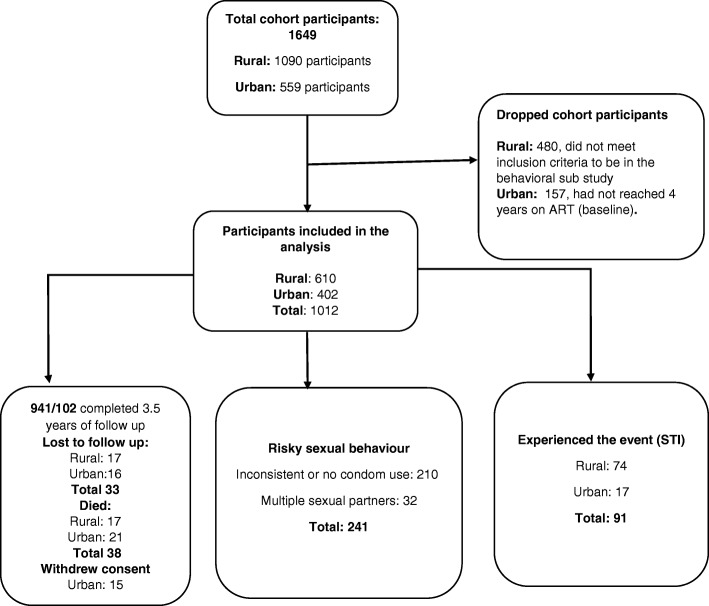
Flow chart showing urban and rural cohort participants follow up information. This figures shows the flow chart of the cohorts participants

Overall, 1012 individuals on ART for 4 years or longer were included in the analysis. Of these, 402 (39.8%) were from the urban cohort and 610 (60.2%) from the rural cohort. Most patients were female (69.6% urban vs 75.7% rural cohort) and about half (53.3% urban vs 54.4% rural) were not married. At baseline, mean age was 42.8 years (SD 8.5). Slightly over a half (53.5%) of the urban but only 35.3% of the rural participants attained secondary education or higher. At baseline, the most common ART regimen was zidovudine, lamivudine, and nevirapine in the rural cohort and a tenofovir-based regimen in the urban cohort. At baseline, almost all participants (~ 90% in both cohorts) had a CD4 > 200 cells/mm^3^, but the mean CD4 count was lower in the urban compared to the rural cohort (mean CD4 cell count 426 vs 564 cells/mm^3^) Table 1**.** Cohort participants contributed a total follow up time of 32,806 person-years with a mean follow up duration of 51.3 months (SD 15.3). Almost all participants (352 or 87.1% of the urban cohort and 589 or 96.6% rural cohort participants) completed 3.5 years of follow up. Sixteen (3.9%) urban and 4 (1.0%) rural cohort participants were lost to follow up or transferred out; Twenty-one (5.2%) urban participants and 17 (2.9%) rural participants were reported dead and 15 urban participants withdrew study consent during follow up. (Fig. 1).

**Table 1:** Baseline characteristics of the cohort participants

Variable	N	Urban (IDI)	N	Rural (TASO)
		N (%)		N (%)
Baseline Age in years	402		610	
18–34		118 (29.3)		52 (8.5)
35–44		180 (44.8)		274 (44.9)
> 45		104 (25.9)		284 (46.6)
Gender				
Female		280 (69.6)		462 (75.7)
Male		122 (30.4)		148 (24.3)
Marital status				
Married/cohabiting		187 (46.5)		278 (46.6)
not married		215 (53.5)		332 (54.4)
Educational level				
No formal education		15 (3.7)		110 (18.0)
Primary Level		173 (43.0)		285 (46.7)
≥ Secondary level		214 (53.3)		215 (35.3)
Employment				
Formal employment		178 (44.3)		105 (17.6)
No formal employment		224 (55.7)		491 (82.4)
Baseline ART regimen				
ZDV 3TC EFV		123 (33.5)		113 (18.5)
D4T 3TC NVP		66 (18.0)		0
ZDV 3TC NVP		0		356 (58.3)
TDF based regimen		177 (48.5)		141 (23.0)
Mean time on ART		8.7 (SD 1.2)		7.0 (SD 0.3)
Baseline Viral load				
Mean log 10 Viral load copies/ml		2.65 (SD 0.34)		1.91 (SD 1.27)
< 1000 cells/ml		385 (96)		501 (82.1)
≥ 1000 cells/ml		16 (4.0)		109 (17.9)
Baseline CD4 cell/mm3				
Mean CD4		426(SD 194.4)		564.3 (284.2)
< 200 cells/mm3		43 (10.7)		54 (8.85)
200–499 cells/mm3		237 (60.0)		213 (34.9)
> 499 cells/mm3		122 (30.4)		340 (55.7)
WHO Staging				
Stage 1		2 (0.5)		25 (4.2)
Stage 2		51(12.7)		243 (41.3)
stage 3		232 (57.7)		205 (34.8)
Stage 4		117 (29.1)		116 (19.7)

During follow up, 94 participants were diagnosed with STIs (39 Vaginal discharge syndrome, 29 genital ulcer disease, 13 urethral discharge syndrome and 13 with Syphilis). Of the 94 diagnosed, 84/1012 (8.3%) had at least one STI recorded in the cohort participants and 10/1012 (0.99%) cohort participants had more than one STI, corresponding to an STI incidence rate of 2.8 per 1000-person-years (95% C1:2.3–3.4). Incidence rate was observed among those practicing risky sexual behavior (4.31 vs 2.51 per 1000 person-years), females (3.25 vs 1.47 per 1000 person-years) and rural cohort participants (3.6 vs 1.38 per 1000 person-years). Table 2.

**Table 2:** Baseline analysis of sexually transmitted infections among study participants

Variable	N	No STIs (*N* = 918)	STIs (*N* = 94)
		N (%)	N (%)
Age	1012		
18–34		153 (90.0)	17 (10.0)
35–44		402 (88.6)	52 (11.5)
> 45		363 (93.6)	25 (6.4)
Gender	1012		
Female		662 (89.2)	80 (10.8)
Male		256 (94.8)	14 (5.2)
Marital status	1012		
Married/cohabiting		422 (90.6)	43 (9.3)
Not married		496 (90.7)	51 (0.3)
Educational level	1012		
No formal education		109 (87.2)	16 (12.8)
Primary Level		422 (92.1)	36 (7.9)
≥Secondary level		387 (90.2)	42 (9.8)
Employment			
Formal employment	998	256 (90.5)	27 (9.5)
No formal employment		648 (90.6)	67 (9.4)
Baseline Viral load	1011		
< 1000 copies/ml		811 (91.5)	75 (8.5)
≥1000 copies/ml		107 (85.6)	18 (14.4)
Baseline CD4	1012		
< 200 cells		90 (90.0)	10 (10.0)
≥200 cell		828 (90.7)	84 (9.2)
Site	1012		
Urban		382 (95.0)	20 (5.0)
Rural		536 (90.7)	94 (9.3)

**Fig. 2:**
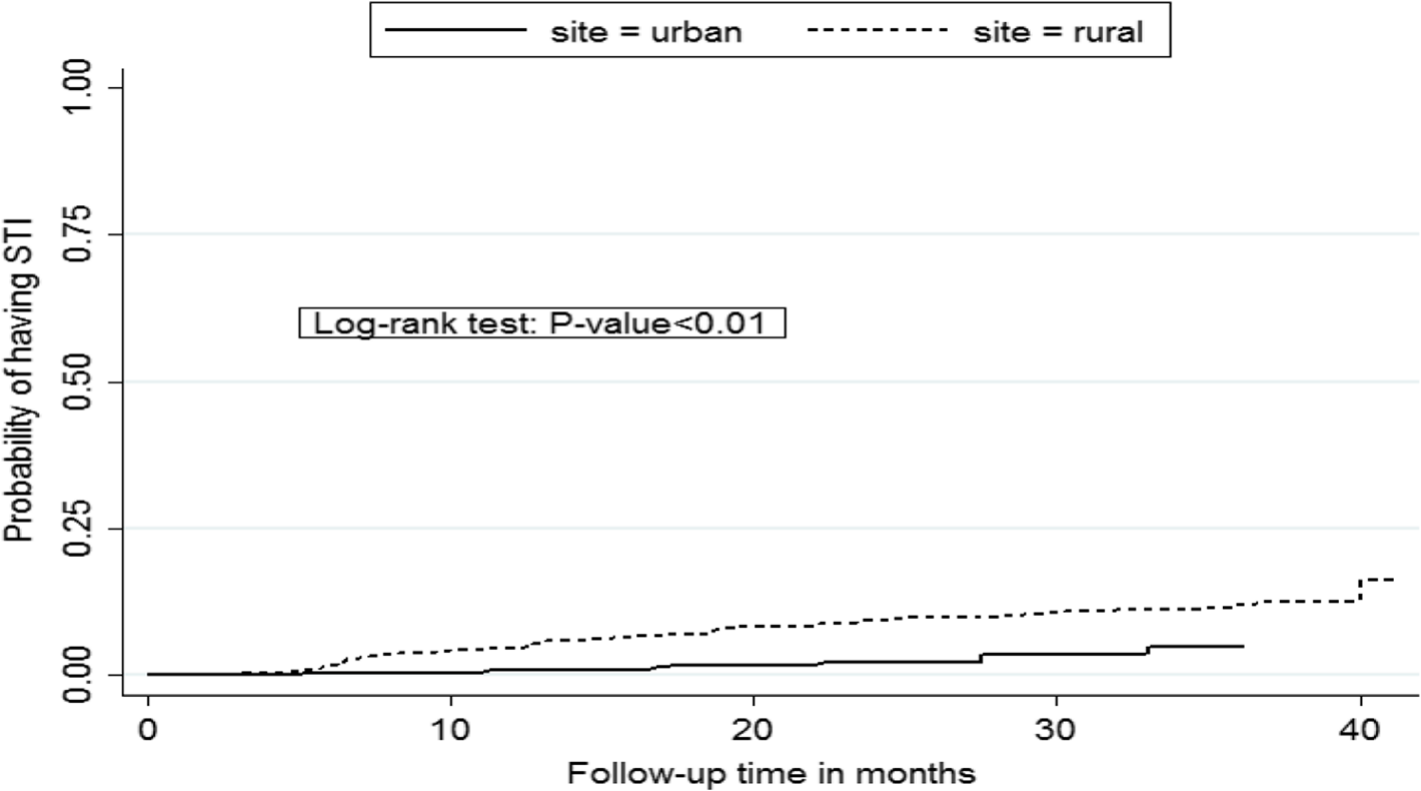
Kaplan-Meier failure plot for cumulative probability of having sexually transmitted infection. In this figure, we observed a significant difference and higher STI incidence over 3.5 years of follow-up in the rural participants compared to the urban participants

In the Kaplan-Meier failure analysis for cumulative probability of having sexually transmitted infections, we observed a significant difference and higher STI incidence over 3.5 years of follow-up in the rural participants compared to the urban participants (log rank test *P* < 0.001). (Fig. 2) In the bivariate Cox model, rural residence (hazard ratio [HR] 2.58, 95% CI: 1.52–4.40), risky sexual behavior (HR 1.70, 95% CI: 1.04–2.77), and younger age (HR 2.13, 95% CI: 1.08–4.21 for 18–34 years and HR 1.73, 95% CI: 1.07–2.83 for 35–44 years as compared to > 44 years) were associated with higher incidence of STIs. Being male (HR 0.45, 95% CI. 0.25–0.81) was associated with a lower incidence of STIs. Rural residence (adjusted HR [aHR] 2.82, 95% CI: 1.60–5.00); and younger age (HR 2.13, 95% CI: 1.08–4.21 for 18–34 years and HR 1.73, 95% CI: 1.07–2.83 for 35–44 years as compared to > 44 years) remained associated with higher incidence of STIs in multivariate analysis. Being male (aHR 0.51, 95% CI. 0.28–0.92) remained associated with a lower incidence of STIs (Table 3).

**Table 3:** Factors associated with incidence sexually transmitted infections among the cohort participants

Variable	Person time	Event	Un-adjusted cox model	Adjusted Cox-model
			HR (95% CI)	HR (95% CI
Site				
Urban	12,305.7	17	Ref	Ref
Rural	20,500.3	74	2.58 (1.52–4.40)	2.82 (1.60–5.00)
Age at baseline				
18–34	5152.6	16	1.72(1.07–3.00)	2.14 (1.08–4.21
35–44	14,661.4	50	1.52 (0.80–1.84)	1.74 (1.05–2.83)
> 45	12,992.0	25	Ref	Ref
Gender				
Female	23,986.5	78	Ref	Ref
Male	8819.6	13	0.45 (0.25–0.81)	0.51 (0.27–0.93)
Marital status				
Married/cohabiting	15,183.3	42	Ref	
Not married	17,622.7	49	1.00 (0.67–1.52)	
Educational level				
No formal education	4032.5	15	Ref	
Primary Level	15,219.0	36	0.61 (0.34–1.12)	
≥Secondary level	13,554.7	40	0.75 (0.42–1.36)	
Employment				
No Formal employment	8827.1	26	Ref	
Formal employment	23,506.0	65	0.98 (0.62–1.54)	
Risky sexual behavior				
No risky sexual behavior	27,895.2	70	Ref	
Risky sexual behavior	4875.6	21	1.70 (1.04–2.77)	1.40 (0.84–2.33)
Baseline Viral load Log viral load			1.04 (0.86–1.25)	
< 1000 copies/ml	28,780.7	72	Ref	
≥1000 copies/ml	4008.6	10	1.04 (0.87–1.26)	1.21 (0.70–2.08)
Baseline CD4 cell/mm3				
< 200 cells/mm3	3132.3	10	Ref	
≥200 cells/mm3	29,673.8	81	0.87 (0.45–1.67)	
Time on ART			0.98 (0.80–1.20)	

## Discussion

In this cohort study of patients on long-term (≥ 4 years) ART, we found an STI incidence rate of 2.8 per 1000 person-years. The lower incidence rate observed in our study could be because we assessed STIs among patients who were already experienced on ART, thus, they received STIs prevention messages during ART initiation and subsequent clinic follow-up. Rural residence, female gender, and younger age were independently associated with a higher STI incidence.

Data on the incidence of STIs in people on ART in sub-Saharan Africa are scarce. In South Africa, one study also found a low incidence rate of STI of 1.29 per 1000 person-years (95% CI 1.24–1.34) in a cohort of 1465 patients followed for 3 years after initiating ART [12] using syndromic diagnoses of STIs. In the South African study, the rate of treatment seeking for new STIs was seven-fold higher (aRR 7.01, 95% CI 4.64 to 10.59) while on ART compared to prior to initiating ART. Due to lack of published data on the STI incidence rate among people on long-term ART, we could not compare our data to other studies in sub-Saharan Africa.

Our findings that rural residence was associated with higher incidence of STIs was also observed in a nationally representative Uganda survey that assessed STI as self-reported STIs and/or associated symptoms in the past 12 months of the general population aged 18–49 [19]. The lower STIs rates observed among the urban participants could be because they continuously received condom promotion messaging and counseling as evidenced by lower condom use (69.5%,146/210) in the rural participants compared to (30.5%, 64/210) in the urban participants at baseline visit) and urban population had greater exposure to HIV prevention messaging.

In the survey of the general Ugandan adult population, having multiple sexual partners was found to be a significant risk factor for STI [9]. In our cohort, participants reporting risky sexual behavior were 50% more likely to have an STI (aHR 1.51), but the association was not statistically significant (95% CI: 0.91, 2.51). Risky sexual behavior has been shown to persist among people on long-term ART [11–14, 29] and may thus place people on long-term ART at continued risk for STIs and HIV transmission to sexual partners.

The association between female gender and younger age and a high incidence of STIs is consistent with other studies in Uganda and South Africa [11, 19–22]. In a South African cohort study of 1465 patients followed for 3 years after initiating ART, being female and younger were associated with higher incidence of clinic visits for STI treatment [12]. Our findings are not surprising as younger people are a sexually active group, tend to have multiple sexual partners, and their knowledge and awareness of safer sexual behavior and practices may be lower compared to older adults. Additionally, females tend to have a better health-seeking behavior in issues relating to STIs than men and many vaginal discharge may not be an STI [30].

Our study had several strengths, including its prospective data collection design, the inclusion of a cohort in both a rural and an urban setting, and a relatively large sample size with long follow up. An important limitation of our study is that biological samples were not collected to confirm an STI diagnosis. As per Uganda Ministry of Health guidelines (adapted from WHO STI’s guidelines), and similar to the situation in most developing countries, majority of the STI diagnoses were made through a “syndromic and case management approach” [16, 21]. The failure of the syndromic approach to detect STI among asymptomatic patients [31] was a limitation to the study. Another limitation is that some participants may have received an STI diagnosis and treatment in a primary care clinic in between the three monthly visits at the HIV care and treatment center. This could have resulted in an underestimation of the STI incidence.

## Conclusion

In this cohort study of patients on long-term (≥ 4 years) ART, we found STIs incidence rate of approximately 3 per 1000 person-years after 3.5 years of follow-up. Rural and younger persons on ART should be prioritized for routine STIs testing in HIV care clinics to enable early diagnosis and management because if diagnosed late and poorly managed lead to serious complications of their own. Furthermore, they enhance sexual transmission of HIV, therefore, making timely and proper STI diagnosis is of paramount importance.

## Abbreviations

ART: Antiretroviral therapy
IDI: Infectious Diseases Institute
MRC: Medical Research Council
TASO: The AIDS Support Organization
WHO: World Health Organization
